# Supplementary material for: Differentiation of Isomeric TAT1-CARNOSINE Peptides by Energy-Resolved Mass Spectrometry and Principal Component Analysis
Source: Molecules. 2025 Feb 12;30(4):853. doi: 10.3390/molecules30040853 (PMC11858179; doi:10.3390/molecules30040853)
Supplement: Supplementary file 1 [file molecules-30-00853-s001.zip › molecules-3410727-supplementary.pdf]

Supporting information

Differentiation of Isomeric TAT1-CARNOSINE Peptides by Energy-Resolved Mass Spectrometry and Principal Component Analysis

Alicia Maroto [1], Olivier Briand [1], Alessia Distefano [1,2], Filiz Arioz [1], Olivier Monasson [3,4], Elisa Peroni [3,4], Giuseppe Grasso [2], Christine Enjalbal [5] and Antony Memboeuf [1,*]

[1] Univ Brest, CEMCA, CNRS, UMR 6521, 29238 Brest, France; alicia.maroto@univ-brest.fr (A.M.); olivier.briand@univ-brest.fr (O.B.); distefano-alessia@libero.it (A.D.); filiz.arioz@univ-brest.fr (F.A.)
[2] Chemical Sciences Department, University of Catania, 95125 Catania, Italy; grassog@unict.it
[3] CY Cergy Paris Université, CNRS, BioCIS, 95000 Cergy Pontoise, France ; olivier.monasson@cyu.fr (O.M.); elisa.peroni@cyu.fr (E.P.)
[4] Université Paris-Saclay, CNRS, BioCIS, 92290 Orsay, France
[5] Univ Montpellier, CNRS, ENSCM, IBMM, 34093 Montpellier, France; christine.enjalbal@umontpellier.fr
* Correspondence: antony.memboeuf@univ-brest.fr; Tel.: +33-(0)2-98-01-61-20

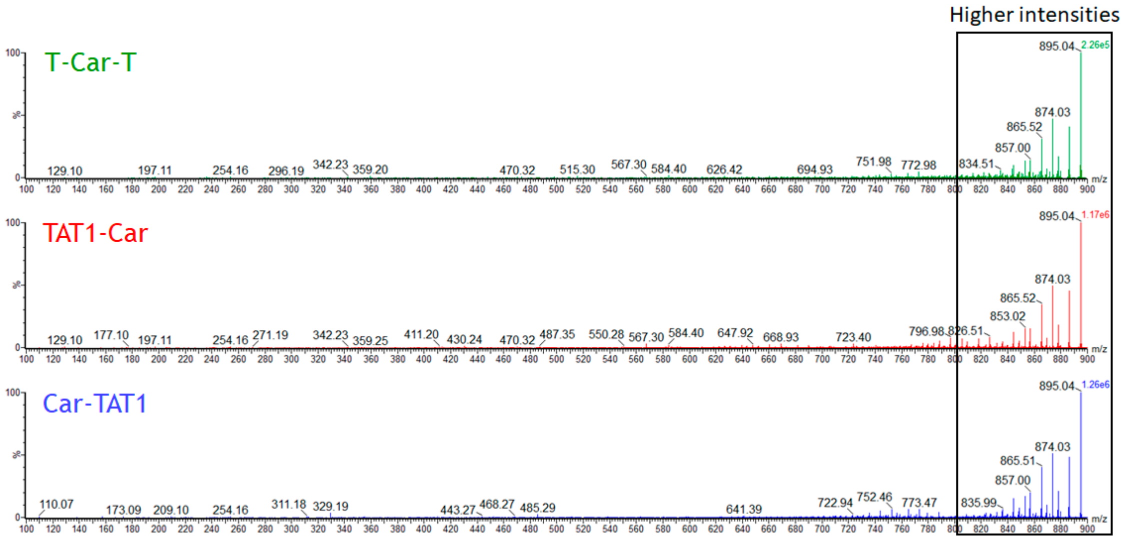

Figure S1. High-resolution MS/MS spectra of T-Car-T, TAT1-Car, and Car-TAT1 at an acceleration voltage of 36 V. The precursor ion at *m*/*z* 895.04 corresponds to the doubly protonated peptide. The highlighted rectangle in the figure marks the 800–900 m/z mass range, where the precursor ion and major fragment ions are concentrated. A magnified view of this region is provided in Figure S2.

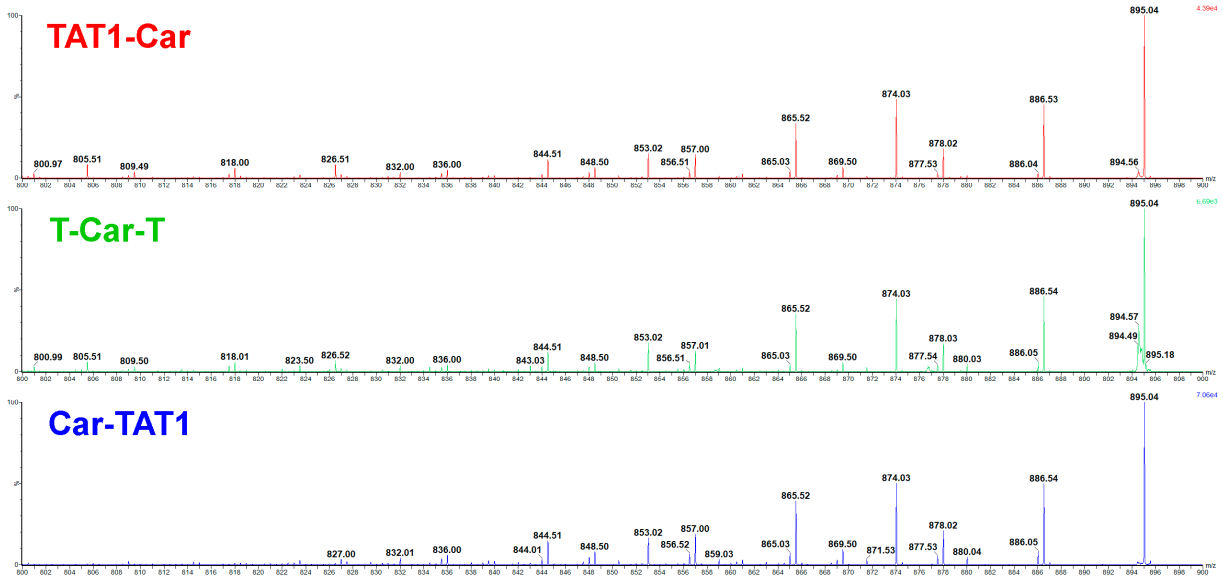

Figure S2. High-resolution MS/MS spectra of T-Car-T, TAT1-Car, and Car-TAT1 at an acceleration voltage of 36 V. The precursor ion at m/z 895.04 corresponds to the doubly protonated peptide. Magnified view of m/z 800-900 from Figure S1.

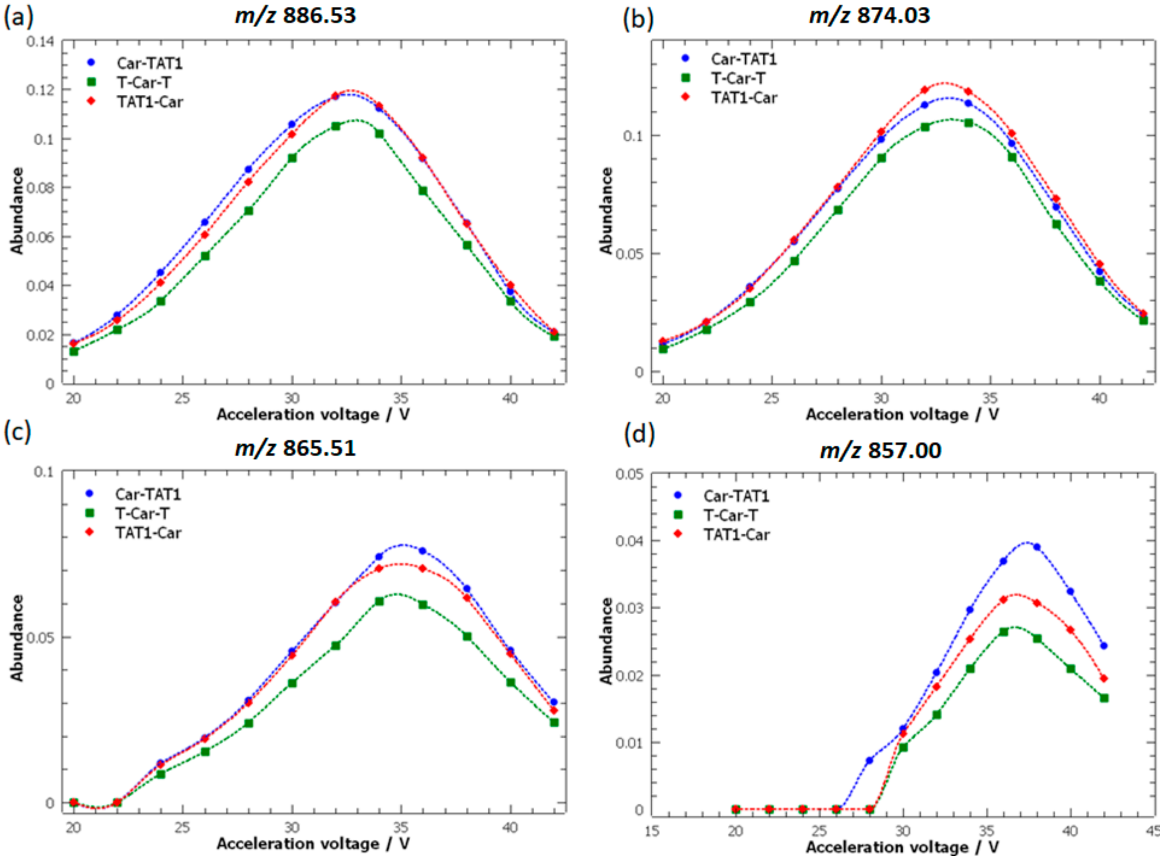

Figure S3. Breakdown curves of the doubly protonated isomeric peptides: Car-TAT1 (blue), TAT1-Car (red), and T-Car-T (green), obtained for the major fragment ions at m/z 886.52, 874.03, 865.51, and 857.00. High-resolution MS/MS spectra were acquired at acceleration voltages ranging from 20 V to 42 V.

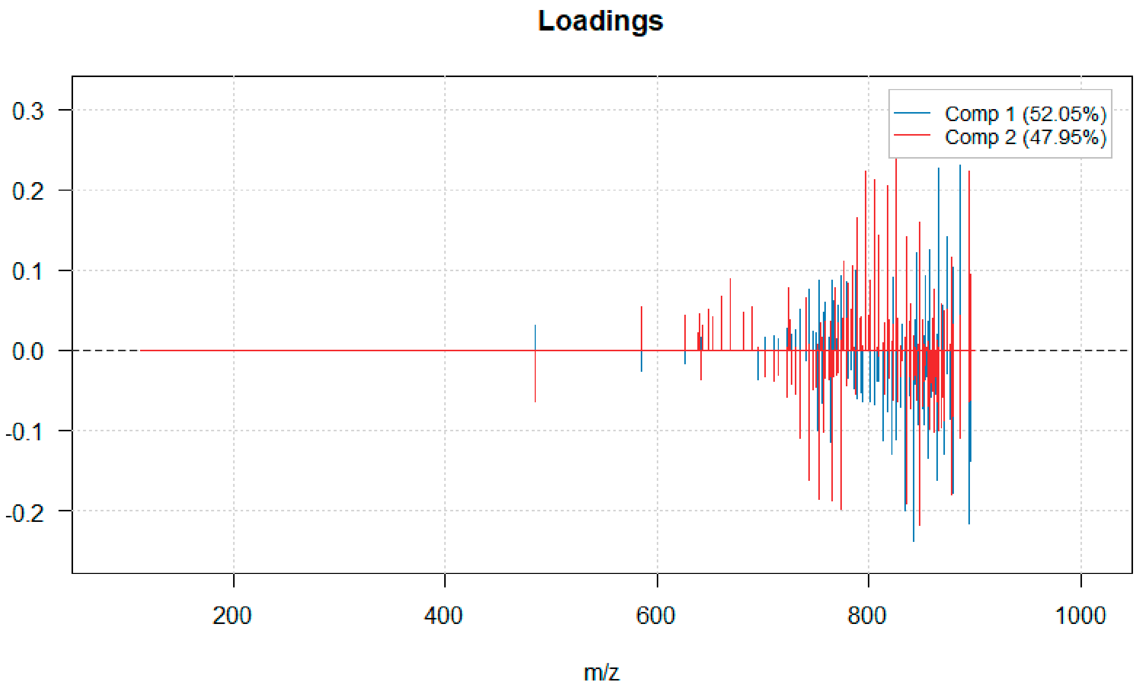

Figure S4. Loadings (line plot). Each line represents the loading of a given m/z value for PC1 (in blue) and PC2 (in red). High positive or negative loadings indicate m/z variables with a greater contribution. The PCA was performed on the MS/MS data between 100 and 900 *m*/*z*.

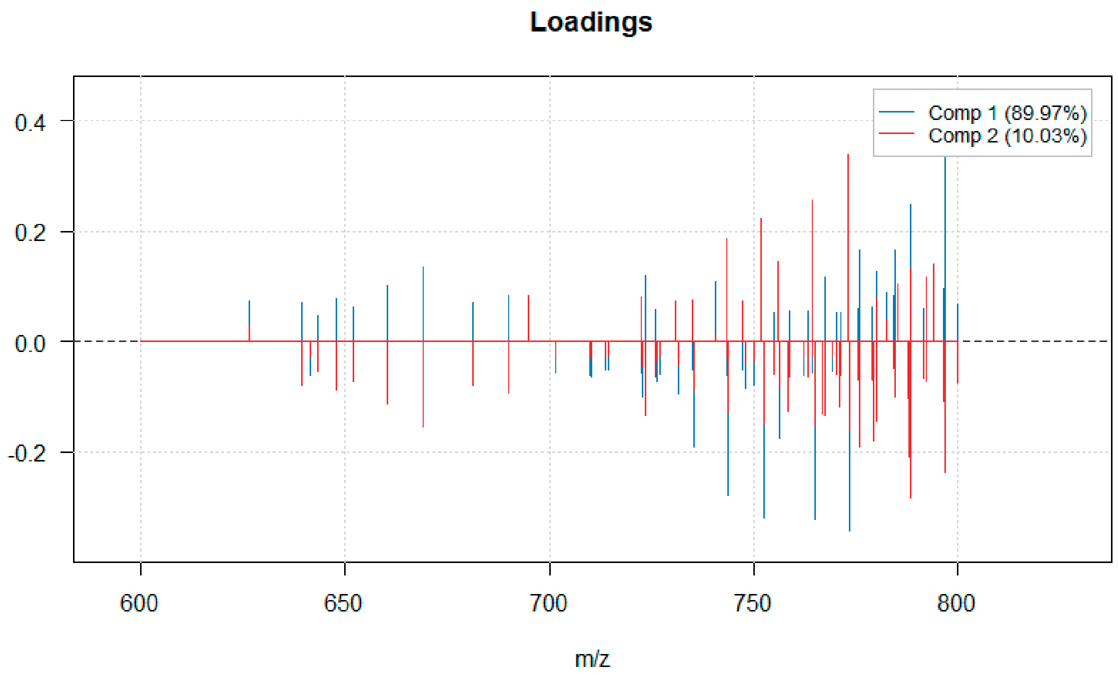

Figure S5. Loadings (line plot). Each line represents the loading of a given m/z value for PC1 (in blue) and PC2 (in red). High positive or negative loadings indicate m/z variables with a greater contribution. The PCA was performed on the MS/MS data between 600 and 800 *m*/*z*.

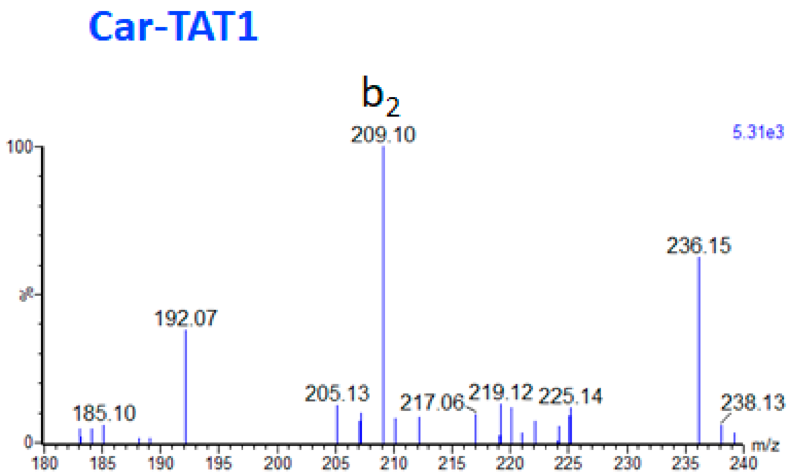

Figure S6. Extract from the high-resolution MS/MS spectrum of Car-TAT 1 corresponding to the b_2 ion.

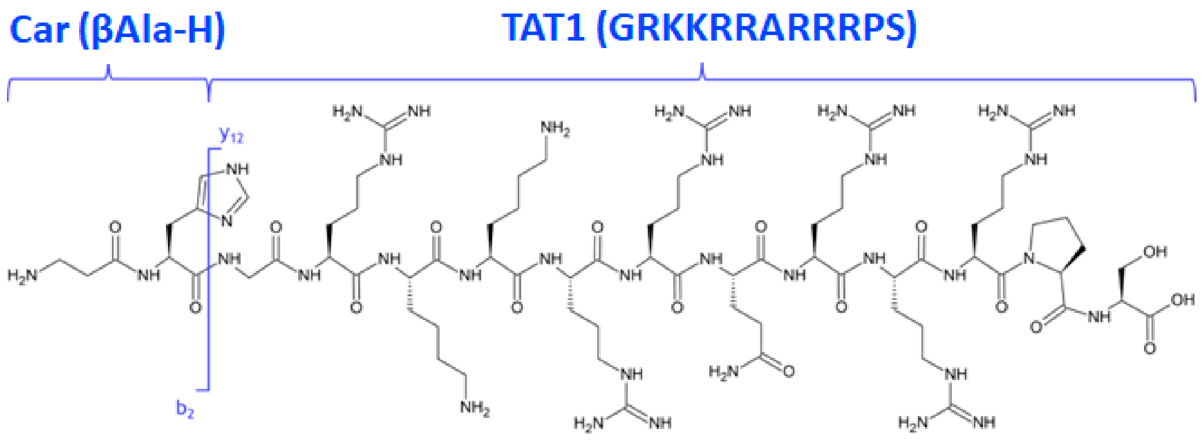

Figure S7. Conventional fragmentation of Car-TAT1 resulting in the complementary fragment ions b_2 and y_{12}.

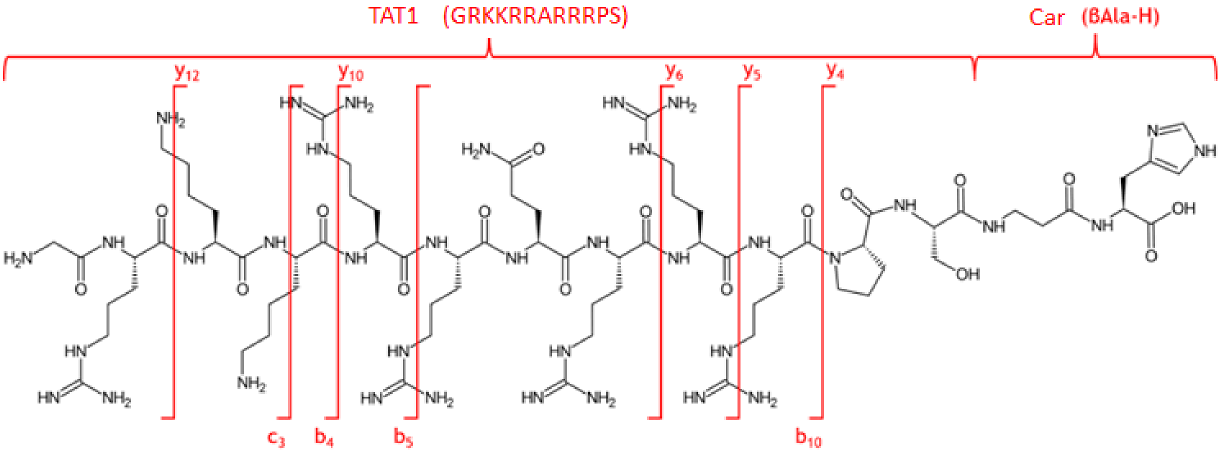

Figure S8. Conventional fragments observed for TAT1-Car.
